# Topical “Soft Candle” Applications for Infected Diabetic Foot Wounds: A Cause for Concern?

**Published:** 2014-06

**Authors:** Shamir O. Cawich, Patrick Harnarayan, Shariful Islam, Bobb Nahmorah J., Steve Budhooram, Shivaa Ramsewak, Michael J. Ramdass, Vijay Naraynsingh

**Affiliations:** 1 Department of Clinical Surgical Sciences, University of the West Indies, St. Augustine Campus, Trinidad and Tobago, West Indies;; 2 Department of Surgery, San Fernando General Hospital, Trinidad & Tobago, West Indies

**Keywords:** Diabetes, Foot Infection, Alternative, Paraffin, Amputation

## Abstract

**Aims:**

There is a cultural barrier to early medical intervention for diabetic foot infections in Trinidad & Tobago, stemming from the strong cultural belief in “soft candle” as effective treatment. We carried out a case-control study to evaluate the outcomes of “soft candle” to treat diabetic foot infections.

**Methods:**

All consecutive patients admitted with diabetic foot infections were interviewed to collect data on: demographics, medical history, unhealthy lifestyle markers (exposure to risk factors for chronic diseases), chosen treatment and details of “soft candle” use. The hospital records were accessed on discharge to records the main outcome measures: HbA_1c_ readings, duration of hospitalization, amputation and in-hospital mortality. Two groups were defined: The control group included patients who sought medical attention after detecting a foot infection. The study group included patients who recognized their infection but voluntarily chose to utilize “soft candle” regimens. We excluded patients who voluntarily chose to use other forms of non-traditional treatment or sought no treatment at all. Outcomes were compared using SPSS ver 19. A two-tailed P value was calculated for variables of interest in each group using Fisher’s exact test. The duration of hospitalization between the groups was compared using paired T-Test. A *P* value <0.05 was considered statistically significant.

**Results:**

There were 442 patients who met inclusion criteria: There were 60 patients in the study group at an average age of 55.2 years (SD ± 11.4; range 43-88): 63% had HBA_1c_ readings >7.0% at presentation and 95% had unhealthy lifestyle habits. There were 382 patients in the control group at an average age of 59.1 years (SD ± 12.6, Range 37-89): 74% with HBA_1c_ readings >7.0% at presentation and 48% with unhealthy lifestyle habits. Patients who used “soft candle” had significantly longer duration of hospitalization (15.5 ± 10.2 vs 9.2 ± 3.9 days; *P*<0.001) and major amputations (13.3% vs 5.6%; *P*=0.048) that was considered clinically significant. There was no difference in minor amputations (31.7% vs 34.3%; *P*=0.770) or in-hospital mortality (1.7% vs 0.52%; *P*=0.355) between the groups.

**Conclusion:**

In its current form, the traditional practice of topical “soft candle” application to diabetic foot wounds may be potentially harmful. Persons with diabetes should be warned about these effects. We have identified the target population for educational campaigns.

## BACKGROUND

Persons with diabetes have between 0.75% (1) and 2% (2) annual risk to develop foot infections. When they occur, diabetic foot infections may have disastrous consequences, including amputation and death.

Early and appropriate medical intervention is needed to reduce the need for amputations from severe diabetic foot infections (3). However, there is a cultural barrier in many developing countries stemming from the strong belief in alternative healers and traditional non-medical therapies. One report from the Caribbean revealed that 29% of patients with diabetic foot infections delayed visits to medical doctors in favour of non-medical therapies, the commonest of which was the use of “soft candle” (4).

This study sought to document the outcomes when topical soft candle applications were used to treat diabetic foot infections. There has been no prior report on the use of this remedy for diabetic foot infections in the world literature. These practices have global importance because they are being carried to developed nations with mass migration. Therefore, health care practitioners in developed countries should also be aware of these practices and their implications.

## METHODS

This study was carried out in Trinidad & Tobago, a twin island nation in the Caribbean with an estimated population of 1,317,714 persons and approximately 15% prevalence of diabetes mellitus in the general population (1). The study was performed across two tertiary referral hospitals with a combined capacity of 1,850 beds to serve a catchment population of 1,050,000 persons across the western half of the Trinidad.

A prospective evaluation of “soft candle” therapy would not be ethical so we carried out an observational study to evaluate its use to treat diabetic foot infections. The local Institutional Review Board granted permission to collect data from all consecutive patients admitted with diabetic foot infections across these institutions from June 2012 to June 2013. Patients were interviewed within 48 hours of admission to collect data on their demographics, prior hospitalization for diabetic foot infections, prior counseling on foot disease, unhealthy lifestyle markers (regular exposure to recognized risk factors for chronic diseases such as smoking, alcohol use or illicit drug use), chosen treatment and details of “soft candle” use (mechanisms of application, route, dose and duration). In order to standardize data reporting, we used the standardized definitions proposed at the 2007 CARICOM Heads of State Government Summit (5): Regular alcohol intake was >1 alcoholic drink daily on 4 or more days per week; regular tobacco use was smoking >1 cigarette daily; and illicit drug use was any reported use of an illegal drug.

We could not justify withholding standard medical therapy from patients in favor of a trial of “soft candle” therapy. Therefore, the second part of this study was strictly observational. We accessed hospital records and recorded three main outcome measures: duration of hospitalization, amputation and in-hospital mortality. We also recorded the glycosylated hemoglobin (HbA_1c_) levels on admission as an index of blood glucose control in the preceding eight weeks.

We defined two groups of patients. The control group included patients who sought medical attention after detecting a foot infection. The study group included patients who recognized their infection but voluntarily chose to utilize “soft candle” regimens. We excluded patients who voluntarily chose to use other forms of non-traditional treatment or sought no treatment at all.

We compared the outcome measures in both groups using Statistical Package for Social Sciences (SPSS) version 19. Descriptive statistics were generated as appropriate. A two-tailed P value was calculated for variables of interest in each group using Fisher’s exact test. The duration of hospitalization between the groups was compared using paired T-Test. A *P* value <0.05 was considered statistically significant.

## RESULTS

There were 695 patients admitted with diabetic foot infections over the study period. We excluded 253 patients who either refused to grant consent to participate in the study, embarked on a trial of non-medical therapy other than “soft candle” preparations or for whom data collection was incomplete. Therefore, the study population included 442 patients: 60 patients in the study group and 382 in the control group (Table 1).

**Table 1:** A comparison of the demographic characteristics of patients in the Control Group and the Study Group

Parameter	Control Group (382)	Study Group (60)

**Male to female ratio**	1.0:1	5.7:1
Men	195	51
Women	187	9
**Age in years**		
Mean ± SD (range)	59.1 ± 12.6 (37-89)	55.2 ± 11.4 (43-88)
**Self described ethnicity**		
Afro-Caribbean descent	143 (37.4%)	34 (56.7%)
East Indian descent	219 (57.3%)	21 (35.0%)
Mixed descent	20 (5.2%)	4 (6.7%)
Chinese descent	0	1 (1.7%)
**Diabetes type**		
Type 1 diabetes	12	0
Type 2 diabetes	370	60
**HBA_1c_ reading at admission**		
Mean ± SD (Range)	7.94% ± 1.54 (4.36-11.23)	7.62 ± 1.61 (4.32-10.5)
**Unhealthy lifestyle markers**		
Alcohol abuse	94	36
Smoking tobacco	88	20
Illicit drug use	2	1

The control group contained 382 patients at a mean age of 59 years. These patients were maintained on oral hypoglycaemic tablets [135], insulin therapy [126] or combination therapy [116] to control their diabetes. Although all patients claimed compliance with their medications, 281 (73.6%) patients had HbA_1c_ readings >7.0% at presentation, indicating poor metabolic control in the preceding 8 week period, and 184 (48.2%) displayed unhealthy lifestyle habits.

The study group contained 60 patients at a mean age of 55 years. These patients were being treated with oral hyoglycaemics [27], insulin [23] or combination therapy [10]. They all claimed compliance to maintenance therapy but 38 (63.3%) had HbA_1c_ readings >7.0% at presentation and 57 (95%) patients had unhealthy lifestyle habits. Table 2 compares the main outcome measures between the groups.

**Table 2:** A comparison of the main clinical outcomes between the control group and the study group

Parameter	Control Group (382)	Study Group (60)	*P* Value	Odds Ratio (95% CI)

Hospitalization (Mean ± SD)	9.2 ± 3.9	15.5 ± 10.2	<0.001	- Continuous variable
Minor amputation	131 (34.3%)	19 (31.7%)	0.770	OR 0.89 (95% CI 0.50 to 1.59)
Major amputation	22 (5.6%)	8 (13.3%)	0.048	OR 2.52 (95% CI 1.07 to 5.95)
Hospital mortality	2 (0.52%)	1 (1.7%)	0.355	OR 3.22 (95% CI 0.29 to 36.05)

**Figure 1:**
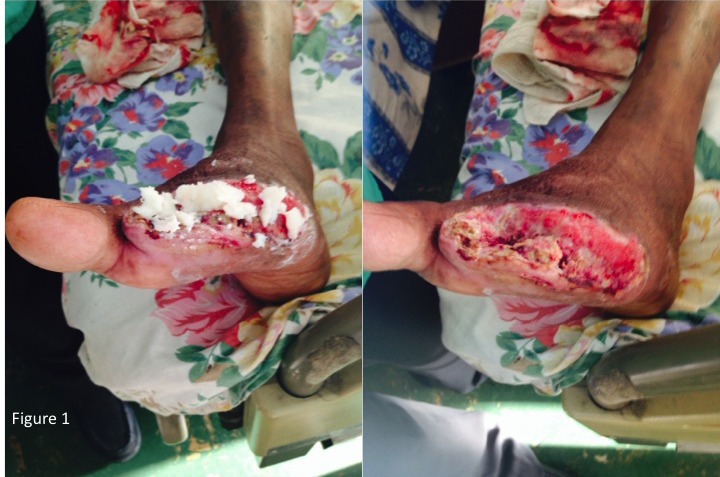
Photograph of an infected diabetic foot wound with soft candle applied topically (1a). The soft candle has been removed and the wound debrided (1b).

When we enquired about the details of therapy, all patients reported that the “soft candle” was heated with an open flame until the wax melted. The hot candle wax was poured directly onto the wound and left in situ. The wound was then covered with a variety of dressings including brown paper bags, banana leaves, saran wrap and conventional gauze swabs. However, there was no consensus on dosage or frequency of applications. Most applied “soft candle” until the wound was “completely covered” and the wax was generally applied once or twice per day (Figure 1).

## DISCUSSION

No attention has been given to the cultural practice of “soft candle” application and its effect on diabetic foot infections in the world literature. This practice deserves attention since persons with diabetes use it to treat skin and soft tissue infections.

Soft candle” is the colloquial name given to container wax candles that were used for several decades in countries with inconsistent or unavailable electricity supply. Although this is no longer the case in most Caribbean urban communities, “soft candle” has persisted on the commercial market driven by a demand for its alleged healing properties.

These candles contain mixtures of paraffin, mineral oil, fragrances, dyes and other ingredients that vary by manufacturer. There are countless anecdotal reports in social media and internet-based resources touting the therapeutic benefits of “soft candle.” We do not contest claims that “soft candle” may have some yet to be elucidated mechanism of action that may prove beneficial, but we found no clinical data in medical literature to substantiate claims that it is useful to treat diabetic foot infections.

More importantly, we demonstrated a relationship between “soft candle” use and worsened outcome measures. There was a significant increase in the duration of hospitalization by 6.3 days when patients opted for a trial of “soft candle” therapy. This has obvious implications on treatment cost and adversely affects the patients’ livelihood since 85% of the users of “soft candle users” were males (more likely to be breadwinners for their families) at an average age of 55 years (still likely to be active in the workforce).

Additionally, “soft candle users” had a significantly increased incidence of major amputations (13.3% vs 5.6%). This is extremely important considering the fact that major amputations lead to reduced quality of life, impaired functionality, depression, social morbidity and increased mortality (3, 6-8).

While the nature of this study does not prove a causal relationship between “soft candle” use and worsened outcome measures, there are plausible mechanisms by which this may theoretically occur.

### Delayed commencement of proven medical therapy

Patients who opted for a trial of “soft candle” therapy voluntarily delayed their presentation to conventional medical practitioners by a mean of 8.1 days (SD 4.6; Range 2-21). The delay in itself may have allowed the infections to progress unchecked without the benefit of proven medical therapies.

### Absence of Content Standardization

Paraffin wax is a firm, odorless, bland, white substance at room temperature. It is one of the commonest types of wax used to make candles. Chandlers typically add mineral oil, fragrances, dyes and other additives to paraffin wax to make their candles more attractive to purchasers. They usually do not disclose the additives because they are considered trade secrets and disclosure is not legally required. Therefore, the contents of “soft candle” vary significantly. This lack of standardization is not acceptable for any other substance whose use is purported for therapeutic value and it should not be acceptable if “soft candle” is to be used for medicinal purposes.

### Potential for Thermal Injury

Although there was no consensus on the dosage and/or frequency of application, “soft candle” was applied in a similar manner by all users. Essentially, the candle is heated on an open flame until the wax melts, the hot wax is poured directly onto the wound and covered by a variety of dressings.

Paraffin wax is a mixture of straight-chain hydrocarbons that are formed as by-products during petroleum distillation. At room temperature, paraffin wax exists in a solid state but its melting point varies widely depending on the ratio of component hydrocarbons (9). There are two types of paraffin wax described: low melt point paraffin has a melting point around 130°F and is used in container candles because it tends to be amorphous at room temperature; high melt point paraffin has a melting point >130°F and is used for pillar candles since it is harder. Because most candles contain both types, the melted wax should have a minimum temperature of 130°F. Additionally, paraffin has a specific heat capacity of 2.5 joules per gram kelvin (10), making it an excellent material to store heat. Application of any material at this temperature to living tissues will generate thermal tissue injury and impair healing.

Burn injuries to the skin have already been reported from hot candle wax (11-15). Diabetics are particularly susceptible to this because they may not be able to appreciate ongoing thermal injuries due to sensory neuropathy. This is compounded by the fact that there is no way for the user to effectively control the temperature of the hot paraffin that is applied to the wound. However, burn wounds may be difficult to diagnose because these patients already have open wounds with infected and ulcerated skin. In our study, we could not find a reliable way to differentiate infected burn wounds and therefore we could not report on this as an individual outcome measure.

### Stimulation of chronic inflammatory response

There are descriptions of medical wax applications as heat therapy for arthritis, myalgia and non-specific pain (16-20), but these are all topical therapies applied to intact skin. Spectroscopy studies on in vivo human skin have revealed that the paraffin / base oil elements do not penetrate deeper than the stratum corneum layer (21-25) and so have no direct tissue-related effects.

However, this is not the case with diabetic foot infections. Because these infections are always accompanied by a break in the skin, the paraffin is exposed to sub-cutaneous tissues and may produce unwanted effects. The medical literature contains several reports of patients who develop intense inflammatory reactions after injections of sub-cutaneous paraffin, mineral oil and Vaseline. Collectively, the term “paraffinoma” has been applied to the clinico-pathologic features of the chronic inflammatory response to these oil based substances (26). There have been several reports of foreign body granulomas, marked desmoplasia and destructive ulcers developing after subcutaneous injections at the male genitalia for penile enlargement (26-33), female breasts for augmentation (34-40), gums with dental implants (41) and lower limbs (42-45). We have encountered no reports of this type of reaction specifically in diabetic foot infections, but this might be difficult to recognize as the skin is already infected and ulcerated. At the very least, “soft candle” applications might be retarding healing due to the chronic inflammatory response it generates.

### Potential Carcinogenic Effect

Paraffin and related base oils are derived as byproducts of petroleum distillation (46). During this process, alkylated polycyclic aromatic hydrocarbons may remain in these products and are potentially carcinogenic (46). These molecules may be released by vaporization and this is the basis behind suggestions that burning candles in enclosed spaces may increase lung malignancies (47). There have also been suggestions that topical exposure to paraffin, mineral oils and associated impurities may be carcinogenic to skin (46-49). Scott *et al* (49) studied the skin lesions that resulted from chronic exposure to paraffin products. He described epitheliomas (paraffin workers’ cancers) that were areas of chronic indurated dermatitis with wart-like appearances. Notably, he reported malignant change arising in one of these lesions (49).

Although there is evidence in early studies that paraffin, mineral oils and associated base oil impurities have carcinogenic properties (46-49) especially with lower levels of refinement (50-52), most authorities point out that modern refinery techniques leave significantly less amounts of alkylated polycyclic hydrocarbons and associated impurities, removing any carcinogenic effects (46). Lu *et al* (53) performed animal experiments on mice pretreated with UV light. They compared mice control mice to mice treated with topical applications of several commercially available crèmes containing paraffin and mineral oils. They noted a significant 69% increase in the incidence of skin malignancies (53). It appears the evidence is still emerging in this area but there have been no studies on the potential carcinogenic effect on open infected wounds in diabetics.

## SIGNIFICANCE OF THE STUDY

Governments across the Caribbean region have recognized that the morbidity from diabetic foot infections is insurmountable (1-4). Therefore, they have developed strategies to limit the consequences of diabetic foot infections (5). In Trinidad & Tobago, there is a well-developed policy to limit the consequences of diabetic foot infections (54).

Dedicated diabetes clinics have been placed in high traffic areas within the community. They are staffed by trained multidisciplinary teams capable of meeting all needs of persons with diabetes and there is a well-developed referral system mandating routine referral to tertiary care hospitals for specialist assessment (54). Additionally, the government of Trinidad & Tobago provides these services at no cost to all persons with diabetes (54). Therefore, the target population has affordable, unrestricted, easy access to these services. The diabetes clinics also coordinate educational campaigns and provide primary care services.

Despite the focus on preventive strategies in Trinidad & Tobago, there has been no focus on the use of “soft candle”, although there are demonstrated ill effects. This suggests that our efforts are not yet optimized, especially since 63% of the “soft candle users” had HBA_1c_ readings >7% at presentation and 95% had unhealthy lifestyle habits.

We have demonstrated that this practice is potentially harmful in its current form. We suggest that educational campaigns should be launched to combat this problem by making the patients aware of these potential ill effects. These campaigns should target middle-aged men with Type 2 diabetes. These men require special attention in any event because they tend to have poor metabolic control despite claims of compliance to therapy. These campaigns have been shown to be beneficial for positive behavior modification^55^ that is important since the majority of these men had poor lifestyle habits.

Campaign messages should convey the need for diabetics to treat foot infections with the appropriate gravity, seeking immediate medical attention instead of home remedies. At the very lease, these campaigns should aim to dissuade soft candle users from this practice or at least to educate them to use medical therapy concomitantly. There should be further research to determine whether this therapeutic method is harmful in itself or whether the outcomes here were due to the delay.

## CONCLUSION

In its current form, the traditional practice of topical “soft candle” application to diabetic foot wounds may be potentially harmful. Patients with diabetes should be warned about these ill effects. We have identified the target population for educational campaigns.

## FUNDING

No funding has been received for this work
